# Sensory dominance and multisensory integration as screening tools in aging

**DOI:** 10.1038/s41598-018-27288-2

**Published:** 2018-06-11

**Authors:** Micah M. Murray, Alison F. Eardley, Trudi Edginton, Rebecca Oyekan, Emily Smyth, Pawel J. Matusz

**Affiliations:** 1 The Laboratory for Investigative Neurophysiology (The LINE), Department of Radiology, and Neuropsychology and Neurorehabilitation Service, University Hospital Center and University of Lausanne, Lausanne, Switzerland; 2 Department of Ophthalmology, Fondation Asile des Aveugles and University of Lausanne, Lausanne, Switzerland; 3 EEG Brain Mapping Core, Center for Biomedical Imaging (CIBM), Lausanne, Switzerland; 4 Department of Hearing and Speech Sciences, Vanderbilt University, Nashville, TN USA; 5 Department of Psychology, University of Westminster, London, UK; 6 Department of Psychology, City, University of London, London, UK; 7 Information Systems Institute at the University of Applied Sciences Western Switzerland (HES-SO Valais), Sierre, Switzerland

## Abstract

Multisensory information typically confers neural and behavioural advantages over unisensory information. We used a simple audio-visual detection task to compare healthy young (HY), healthy older (HO) and mild-cognitive impairment (MCI) individuals. Neuropsychological tests assessed individuals’ learning and memory impairments. First, we provide much-needed clarification regarding the presence of enhanced multisensory benefits in both healthily and abnormally aging individuals. The pattern of sensory dominance shifted with healthy and abnormal aging to favour a propensity of auditory-dominant behaviour (i.e., detecting sounds faster than flashes). Notably, multisensory benefits were larger only in healthy older than younger individuals who were also visually-dominant. Second, we demonstrate that the multisensory detection task offers benefits as a time- and resource-economic MCI screening tool. Receiver operating characteristic (ROC) analysis demonstrated that MCI diagnosis could be reliably achieved based on the combination of indices of multisensory integration together with indices of sensory dominance. Our findings showcase the importance of sensory profiles in determining multisensory benefits in healthy and abnormal aging. Crucially, our findings open an exciting possibility for multisensory detection tasks to be used as a cost-effective screening tool. These findings clarify relationships between multisensory and memory functions in aging, while offering new avenues for improved dementia diagnostics.

## Introduction

Healthy aging has a pervasive impact on the structure and the function of the brain, although there are differential effects across cortical regions, neuronal networks and neurotransmitter systems. Age-related changes characteristically have a negative impact on sensory, perceptual, emotional and higher-order, memory-related and executive functions^1–5^. Yet, healthy older adults typically show an enhancement in the gain resulting from multisensory integration (MSI) of redundant cross-sensory (but not unisensory) information compared to younger adults^6–8^. MSI is known to provide a wide range of benefits across both behaviour and neural processing^9^. Additionally, there is considerable evidence that sensory and multisensory abilities invariably scaffold cognitive abilities^10,11^. In stimulus detection, MSI improves detection speed over and above mere probability summation^12^, with these enhancements associated with non-linear interactions within low-level sensory cortices at early, pre-attentive brain processing stages^13,14^. To provide much-needed clarification how such low-level MSI change across the lifespan^10,11^, here we assessed links between them and 1) low-level attributes such as sensory dominance, and 2) high-level functions such as memory and its impairment in pathological conditions.

In older adults, enhanced multisensory processing may be a by-product of decreased efficiency at filtering distractions and sensory noise^15^, rather than a consequence of general cognitive slowing and by extension increased relative gains from multisensory information. Paradoxically, as top-down inhibitory processes become less efficient at filtering distractions and sensory noise with age, the failure to suppress processing in task-irrelevant senses could result in enhanced multisensory processing in the elderly^6,16^. Because the effortful, top-down inhibitory processes are thought to be cholinergically mediated^17^, they are particularly vulnerable to the cholinergic *decline* associated with healthy aging, as well as to the cholinergic *disruption* that characterises neurodegenerative conditions, such as Mild Cognitive Impairment (MCI) and Alzheimer’s disease (AD). It is important to note that such a link between cholinergic activity and multisensory processes has been established in rodents^18^, with a particularly prominent role in multisensory memory formation and retrieval^19,20^. Thus, disruptions in cholinergic processing likely contribute to further alterations in multisensory processing during abnormal aging, which could potentially have diagnostic value, if gauged appropriately.

MCI is characterised by isolated changes in cognitive function that go beyond those expected in healthy aging. While it remains unclear whether MCI and AD are distinct or sequential, related disorders^21^, the likelihood of developing dementia, including AD, is considerably raised in both amnestic and regular MCI (49% and 27%, respectively^22^). Although MCI and AD are defined primarily by episodic memory loss, they can be accompanied by disruptions in top-down, goal-related attentional control functions, with some purporting for these top-down attentional impairments to be driving the deficits in memory function^23^. More recent accounts suggest that disruptions in more general, neuromodulatory inhibitory functions are responsible for both deficits^17^. This pattern, combined with cognitive reserve in older individuals, has rendered diagnosis of MCI and AD particularly challenging.

As such, early identification of individuals at-risk for MCI (or AD) remains a major public health issue^24^. Therefore, integrating multiple sources of diagnostic information is commonplace. Currently, MCI diagnoses are based on the Mini-Mental State Examination (MMSE) and performance on memory and executive function tasks including specific measures on the Hopkins Verbal Learning Test (HVLT)^25^, often requiring additional assessment of activities of daily living for confirmation. Challenges pertinent to the use of these tools are the fact that the materials are sold and copyrighted by a company and the need for trained clinicians to administer and score them^26^. As such, they require a clinical infrastructure and consequently are time- and resource-consuming (for both the patients and clinicians). Many argue that MMSE is more suited for capturing differences in more general functions, rather than more specific memory-related functions^27^. An additional consideration, however, is that intellect and/or cognitive reserve (as well as other comorbidities) of the patient can bias performance on these two tools. This is further compounded by influences of tester reliability, particularly on the MMSE, leading some authors to suggest that the MMSE may be a “less practical solution for many clinicians”^26^. Multisensory perception tasks may offer a particularly promising addition to these screening assessment tools.

The unique potential utility of multisensory perceptual tasks hinges on several advantages: (1) they are quantitative measures robust to being administered in the home environment and by persons without specialist clinical training or even autonomously, (2) as such, they can be administered with minimal time and resource demands for both the patient and medical staff, and (3) growing literature has demonstrated group differences in multisensory processing as well as sensory dominance in such multisensory tasks between both healthy young and healthy aging individuals, as well as those healthily and abnormally aging.

On the one hand, initial evidence points to conserved, albeit reduced, multisensory benefits in MCI compared to healthy aging^28^. However, a strong contribution of compensatory, attention-based processes^16^ to these benefits cannot be excluded. On the other hand, sensory dominance has been demonstrated across healthy young and older in multisensory tasks^6,29^. Sensory dominance describes a situation where in a given context, whether it is a task or a specific individual, input into one sense is processed preferentially (e.g., faster or more accurately) than inputs into other senses. Sensory dominance is known to influence the magnitude of multisensory benefits and how they are generated by the brain^30,31^. Considering the disruptive influence of age-related neurodegenerative conditions such as MCI and AD on visual and auditory systems, sensory dominance patterns are likely clinically important^32–34^. Aging may push individuals towards being typically more visual/auditory, which in turn may modulate the magnitude or even presence of benefits in multisensory perception tasks. Existing, scant studies employing multisensory detection tasks showed more^29,35^ or less explicitly^6^ that healthy older individuals might be overall visually dominant. Yet, disparities in inclusion criteria (e.g., differences in the employed screening tests and cut-offs) and lack of group-wise quantitative assessment of frequency versus strength of the sensory dominance patterns necessitate more systematic investigations. Examining such group differences, in both uni- and multisensory processing might provide clinicians with a valuable assessment tool in dementia screening and perhaps diagnosis. Subtle differences, across one or both perceptual measures, might constitute particularly sensitive markers for preclinical decline and as such provide a differential diagnosis between neurodegenerative conditions and healthy aging. In contrast to traditional, questionnaire-based tools, they may do so with little time and resource demands on patients and medical personnel. Thus, investigations into patterns of perceptual processing in healthy and at-risk individuals are crucial.

The present study aimed to assess differences in unisensory and multisensory processing across healthy young, healthy older adults and older adults with MCI. A closely related aim was to explore the potential discriminative value of these perceptual processes as a measure of preserved versus impaired cognitive function. We capitalise here on prior findings in healthy adults, linking multisensory benefits in well-researched, simple detection tasks with sensory dominance on the one hand, and memory functions on the other^10,36^. Such tasks minimise the confounding influence of compensatory attentional processes on multisensory processing. Our predictions were that our task will demonstrate group differences in the strength of multisensory benefits, and frequency/strength of relative sensory dominance patterns^6,28,29^, and also that unisensory dominance patterns and multisensory benefits would be associated^30,31^. More importantly, based on previous work in healthy and abnormally aging older adults, we predicted that the strength of multisensory benefits and/or the strength of relative unisensory (auditory or visual) dominance measured from a simple detection task will reliably distinguish in ROC (Receiver Operating Characteristic) analysis between individuals with preserved versus impaired cognitive functioning, as assessed by the MMSE. While a battery of neuropsychological tests, including HVLT, was used to guide our older-participant group assignment (as either healthy or with MCI), the score threshold as well as the clinical sensitivity ROC analyses were determined using the MMSE score due to the more general nature of the cognitive functioning assessed by this tool.

## Results

Overall, the groups were equated on general characteristics and baseline cognitive performance (Table 1). While there was a significant IQ difference across groups (F_(2,97)_ = 5.69; p = 0.005; η_p_^2^ = 0.11), this was driven by the difference between healthy young (HY) and healthy older (HO). There was no reliable difference between HO and MCI (p = 0.07) or between HY and MCI (p = 0.99). There were no reliable differences in years of education (F < 1). There were more women in the HY than HO or MCI group; the latter two of which did not differ. Across neuropsychological tests, the MCI group expectedly demonstrated poorer learning and memory scores than the other two groups (Table 1 and Supplemental Results).

**Table 1:** Participant demographics with standard deviation indicated in parentheses.

	Healthy Young	Healthy Older	MCI
Number of included participants (number of excluded participants)	47 (4)	35 (14)	18 (5)
Gender	21% Male	40% Male	50% Male
Age (years)	22.0 (3.9)	72.7 (6.2)	76.9 (8.4)
Handedness	47 Right-handed 0 Left-handed	31 Right-handed 4 Left-handed	16 Right-handed 2 Left-handed
Years in Education	15.8 (2.7)	16.7 (4.8)	17.1 (5.3)
NART Full scale IQ	116.9 (4.7)	120.7 (4.9)	116.9 (7.9)
MMSE	n.a.	29.2 (0.6)	24.2 (2.1)

**Figure 1:**
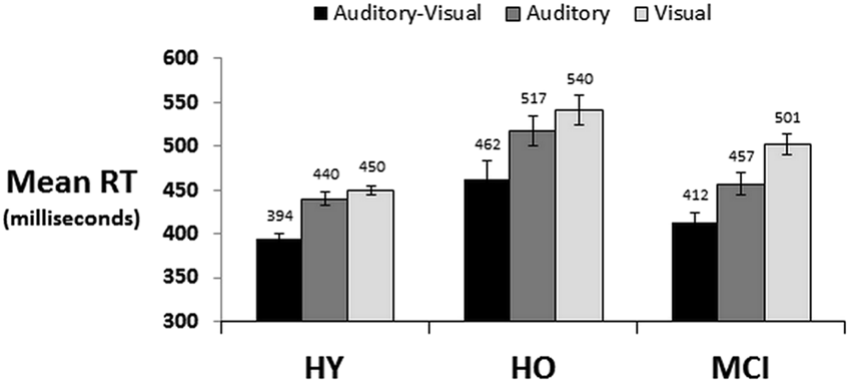
Mean reaction times (standard error of the mean indicated) of the three groups on the multisensory detection task. (HY = healthy young, HO = healthy older, MCI = mild cognitive impairment).

All participants readily completed the detection task with high accuracy (see Supplemental Results). Reaction times from the detection task were first analysed with a 3 × 3 mixed-model ANOVA using stimulus condition (AV, A, and V) as the within-subject factor and population (HY, HO, and MCI) as the between-subjects factor (Fig. 1). Results are reported using the Greenhouse-Geisser correction for violation of sphericity, when appropriate, along with corrected degrees of freedom. Post-hoc t-tests entailed the Bonferroni-Holm correction for multiple comparisons. There was a main effect of stimulus condition (F_(1.5,145.9)_ = 207.41; p < 0.001; η_p_^2^ = 0.68), with generally faster RTs for the AV than either the A or V condition, which likewise significantly differed (all p’s < 0.001). There was a main effect of group (F_(2,97)_ = 13.72; p < 0.001; η_p_^2^ = 0.22) with generally slower RTs in the HO than either HY (p < 0.001) or MCI (p = 0.038), the latter two of which did not generally differ in overall RTs (p = 0.37). Finally, there was a significant 2-way stimulus condition × population interaction (F_(3,145.9)_ = 5.26; p < 0.002; η_p_^2^ = 0.10). Before we evaluated this interaction with a series of separate follow-up 1-way ANOVAs for each population, we tested whether unisensory processing differed across age groups with a 2 × 3 mixed model ANOVA using stimulus condition (A and V) as the within-subject factor and population (HY, HO, and MCI) as the between-subjects factor. There was main effect of stimulus condition (F_(1,97)_ = 31.80; p < 0.001; η_p_^2^ = 0.25) as well as a significant condition × population interaction (F_(2,97)_ = 4.49; p = 0.014; η_p_^2^ = 0.09). This follow-up analysis has confirmed that there was indeed a statistically reliable change in the sensory dominance patterns across the three groups.

In the follow-up 1-way ANOVA for HY, there was a main effect of stimulus condition (F_(1.3,59.3)_ = 106.9; p < 0.001; η_p_^2^ = 0.70) that was due to faster RTs to AV stimuli than to either A or V stimuli (both p’s < 0.001); the latter of which did not reliably differ (p > 0.15). This indicates that despite no explicit effort on our part to titrate stimulus efficacy, there was no evidence for differences in sensory processing in the HY. In the HO there was again a main effect of stimulus condition (F_(1.4,46.5)_ = 71.7; p < 0.001; η_p_^2^ = 0.68) that was due to faster RTs to AV stimuli than to either A or V stimuli (p’s < 0.001). In contrast to the HY, HO individuals were generally faster to respond to A than V stimuli (p = 0.03). In the MCI individuals, there was a similar pattern. There was a main effect of stimulus condition (F_(2,34)_ = 50.0; p < 0.001; η_p_^2^ = 0.75) that was due to faster RTs to AV stimuli than to either A or V stimuli as well as significantly faster RTs to A than to V stimuli (all p’s < 0.001).

**Figure 2:**
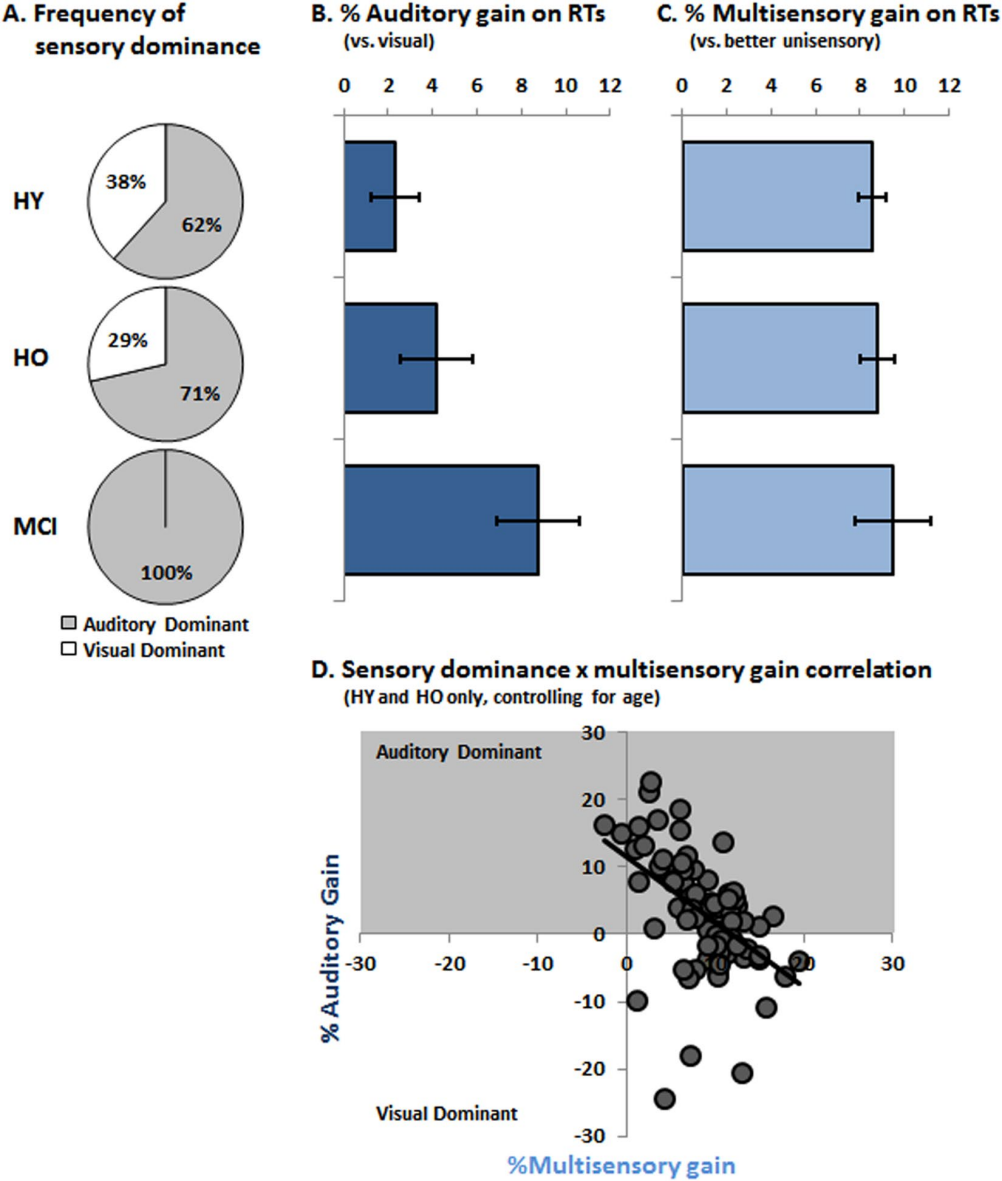
Interplay of sensory dominance and multisensory gains. (**A**) The percentage of individuals exhibiting auditory vs. visual sensory dominance are displayed for each group. (**B**) The strength of auditory dominance was calculated as the percentage difference between unisensory visual and auditory reaction times for each individual. (**C**) The strength of multisensory gain was calculated as the percentage difference between the multisensory and better unisensory condition. In B and C mean values within each group are displayed (s.e.m. indicated). (**D**) The scatterplot shows that the strength of auditory dominance and strength of multisensory gain were inversely correlated across HY and HO groups after controlling for age.

The above analyses would suggest that multisensory benefits are present in all groups, and that there is a shift in unisensory processing with healthy and abnormal aging such that RTs to sounds were significantly faster than those to flashes. This was not the case in HY for whom unisensory RTs were indistinguishable. Moreover, auditory/visual dominance, defined as faster unisensory RTs in each participant^30^, was assumed and confirmed to be equally distributed in HY (χ^2^ = 2.57; p > 0.10) (Fig. 2A). By contrast, there was a significant shift for a propensity of auditory dominant individuals with healthy (χ^2^ = 6.43; p < 0.01) as well as abnormal aging (χ^2^ = 18.0; p < 0.0001); the latter of which were exclusively auditory dominant (Fig. 1A). The distribution of sensory dominance likewise differed between HO and MCI individuals (χ^2^ = 7.20; p < 0.008).

We next quantified each participant’s relative auditory dominance as the difference in mean RT between visual and auditory conditions divided by the mean RT to the visual condition, yielding a percentage of auditory dominance (Fig. 2B). These values significantly differed across groups (F_(2,97)_ = 3.79; p = 0.026; η_p_^2^ = 0.07). Post-hoc contrasts showed that relative auditory dominance was significantly larger in MCI individuals vs. HY (p = 0.007) and showed a non-significant trend vs. HO (p = 0.064); the latter two of which did not significantly differ (p > 0.30). This pattern reinforces the observation of altered patterns of unisensory processing in individuals with MCI. Not only are MCI individuals more often auditory dominant, but the average RT difference between auditory and visual processing is also significantly larger.

Given this evidence, we then quantified each participant’s relative RT benefit from multisensory stimuli vs. their better unisensory stimulus condition (i.e. each participant’s dominant sensory modality), yielding a percentage of multisensory gain (Fig. 2C). This is a common metric of multisensory enhancement^37^. These values did not significantly vary across groups (F_(2,97)_ < 1; p > 0.80; η_p_^2^ = 0.004). We also considered this metric separately as a function of an individual’s sensory dominance (see Supplemental Results and Supplemental Fig. 1). While percentages of multisensory gain were generally larger in visually dominant participants (at least in the HY and HO where both types of dominance were observed), there was no reliable difference across HY, HO, and MCI groups. Multisensory gains were likewise examined for deciles along the RT distribution, by applying Miller’s race model inequality^12^ (Supplemental Materials and Supplemental Fig. S1). Multisensory gains over the fastest 30% of RTs were larger in HO than HY who were also visual dominant, whereas no differences in multisensory gains were observed across HY, HO, and MCI groups who were also auditory dominant. Thus, sensory dominance appears to contribute to age-related differences in multisensory processing, at least in healthy individuals. Moreover, we observed a robust negative correlation in healthy individuals (i.e. HY and HO pooled together) between the relative multisensory gain and the relative auditory dominance, even when controlling for age (r_(79)_ = −0.491; p < 0.001) (Fig. 2D). In other words, larger multisensory gains were observed when sensory dominance was smaller, in line with previous findings^38^. Interestingly, however, this correlation was reliable only for auditory dominant participants (r_(51)_ = −0.699; p < 0.001), but not for visually dominant participants (r_(25)_ = 0.319; p > 0.10). A similar negative relationship between these indices was observed in the MCI group (r_(16)_ = −0.613; p < 0.007). For the MCI group, we additionally observed that the relative multisensory gain correlated positively with the MMSE score (r_(16)_ = 0.532; p = 0.023). No such relationship was observed with any subtest score on the HVLT (all p’s > 0.20). Finally, we also considered how multisensory gain and relative auditory dominance related to neuropsychological assessments (the HVLT and NART). While multisensory gain was positively correlated with NART scores (all p’s < 0.003, there were no reliable correlations with any HVLT score (all p’s > 0.58). By contrast, relative auditory dominance was negatively correlated with the HVLT scores (all p’s < 0.035 with the exception of the retention score, p = 0.30), but showed no correlation with NART scores (all p’s > 0.53).

**Figure 3:**
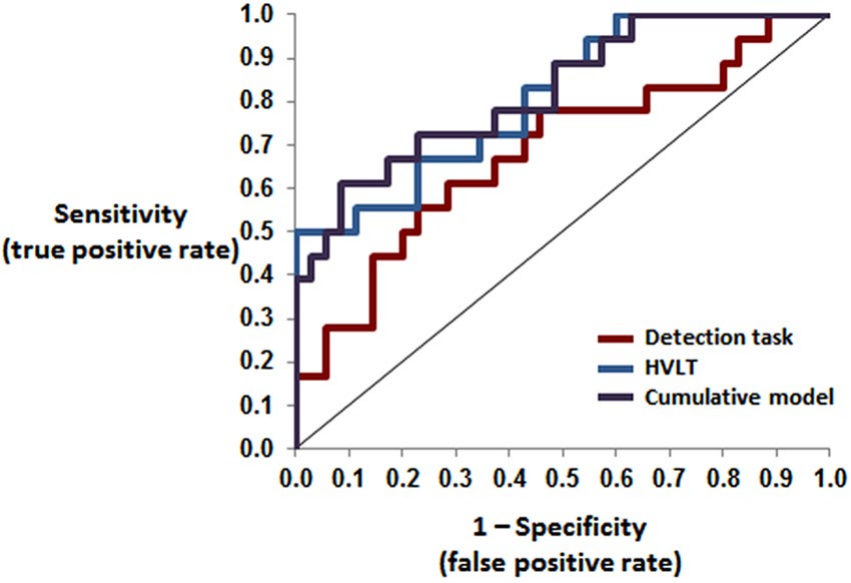
Area under the ROC curve for the classification of MCI vs. HO based on hierarchical logistic regression analyses. The blue line displays the ROC analysis based on a linear combination of the HVLT scores. The red line displays the ROC analysis, based on a linear combination of auditory dominance and multisensory gain. The purple line displays the ROC analysis based on the linear combination of the HVLT and detection task. Specific values are detailed in Table 2.

We next assessed whether accurate classification of HO vs. MCI status, as based on the MMSE score, could be achieved using the above indices of sensory dominance and multisensory processing. For this purpose, we conducted a ROC curve analysis, the gold standard in evaluating diagnostic potential of a given measure. As visible in Table 2, when either relative auditory dominance or multisensory gain was used alone as a classification parameter, the area under the curve (AUC) was not reliably different from chance. However, when the two measures were linearly combined into a single parameter, the AUC was reliably different from chance (p < 0.03; Fig. 3). ROC analyses conducted for each of the four subscales of HVLT, showed, as expected, AUCs reliably larger than chance, all *p*’s < 0.05 (see Table 2). Notably, the AUC value obtained using sensory dominance and multisensory gain was *de facto* within the range of values observed using any of the HVLT subscales or their combination. Finally, we compared in a hierarchical manner the AUC for the combined HVLT subscales, followed by the AUC for the detection task, and then the cumulative model based on both the HVLT and the detection task (Table 2 and Fig. 3). The detection task did not significantly increase the AUC beyond that achieved with the combined HVLT subscales. This suggests that the detection task provides complementary discriminatory evidence that is similar to that achieved with instruments such as the HVLT.

**Table 2:** The Area Under the Curve (AUC) values and their statistical significance for each of the predictors, including multisensory gain and sensory dominance and a model combining the two, as well as each of the four employed Hopkins Verbal Learning Test (HVLT) subscales.

	AUC	Std. Error	*p*-value (vs. chance)	*Asymptotic 95% confidence interval*	
				Lower bound	Upper bound
Sensory dominance	0.616	0.079	0.170	0.412	0.820
Multisensory gain	0.565	0.090	0.441	0.333	0.797
Detection task (combined model)	0.684	0.080	0.029	0.527	0.841
HVLT Learning	0.731	0.077	0.006	0.340	0.928
HVLT Delayed Recall	0.775	0.071	0.001	0.593	0.956
HVLT Retention	0.675	0.082	0.038	0.463	0.887
HVLT Recognition	0.763	0.069	0.002	0.586	0.941
HVLT (combined model)	0.811	0.062	0.000	0.690	0.932
Cumulative model	0.822	0.061	0.000	0.666	0.979

## Discussion

While information processing takes place in environments commonly multisensory in nature, the existing literature is currently unclear as to how healthy and abnormal aging influence this processing. To address this uncertainty, we compared healthy young, healthy old and individuals with MCI on a simple, multisensory detection task involving ostensibly meaningless stimuli, which minimised the confounding influences of compensatory top-down processes on the observed results. By linking multisensory benefits within the sensory profiles present in each of the three groups, we provided novel insights into the influences of both healthy and abnormal aging on basic multisensory functions. The most exciting finding of our study was the demonstration that the relative multisensory gains together with the relative sensory dominance correctly determined participants’ MCI diagnosis, as assessed with the traditional diagnostic test. This may provide a particularly cost-effective and accessible complement to existing screening approaches.

Notably, these findings required scrutiny of both sensory and multisensory processing profiles that were not otherwise visible in general analyses. First, and overall, our RT results demonstrated that low-level multisensory processing is generally preserved in aging (Fig. 2C); both healthy and abnormal (here, MCI). Second, by examining multisensory benefits within each unisensory dominance subgroup, we were able to demonstrate that enhanced multisensory benefits associated in the literature with healthy aging are *de facto* restricted to visually-dominant HO individuals; these individuals were proportionally less frequent among HO individuals. Visually dominant HY and HO groups both showed stronger multisensory RT gains than their auditory-dominant counterparts. Only race model violation analyses within the visually-dominant individuals showed age-related enhancements in multisensory RT benefits^6^. Third, multisensory RT benefits were indeed preserved in MCI individuals, and comparable in magnitude compared to those in HY and HO individuals. However and in a marked contrast to HO individuals, all of the MCI individuals were auditory-dominant. Notably, these findings are in strong agreement with elevated visual thresholds in MCI, compared to healthy individuals in a study^39^ that assessed distinct parameters of visual processing derived from Bundensen’s theory of visual attention^40,41^. We extend these results by demonstrating that MCI seemingly eliminates the incidence of visual dominance in older individuals, which seems to be linked in turn with the presence of enhanced age-related multisensory benefits in this population. More generally, our data also indicate that multisensory gains are negatively correlated with sensory dominance (Fig. 2D); a point to which we return below.

The pattern of our findings is noteworthy as visual and auditory responses were statistically indistinguishable in the HY group, where the absence of aging-induced changes in sensory dominance can be assumed, even though the stimuli were not overtly and explicitly equated in terms of either physical or perceptual features. As auditory responses were faster in the majority of healthy older individuals as well as all MCI, our findings suggest that aging, and perhaps abnormal aging in particular, “pushes” individuals towards auditory dominance. As such, our study enriches the previous, scarce findings in this area. In a study that tested both healthy older and MCI in a detection task with simple stimuli^28^, MCI showed no sensory dominance and healthy older individuals showed visual dominance. However, there was no “baseline” healthy young adult comparison group, and the study promoted the involvement of compensatory processes by using two additional demanding selective-attention tasks. Visual dominance in healthy older versus young individuals was suggested by another study that employed a detection task, this time involving object stimuli^29^, the processing of which has been shown previously to be a visually-dominant^42^. However, the lack of full analyses of RTs in the Diaconescu *et al*. study precludes distinguishing between sensory dominance in lower- versus higher-level perception. Visual dominance was equally suggested by the seminal detection study by Laurienti *et al*.^6^; but, as in the studies discussed above, sensory dominance profiles were not analysed within young or older individuals in terms of incidence, strength or their links with multisensory RT benefits. Thus, one potential implication for future studies in this area is the benefit of including a control group of healthy adults, to treat them as a “normative” group to verify that the employed visual and auditory stimuli are approximately matched in their physical and/or perceptual features. In studies involving more naturalistic stimuli representing real-world objects^29^, where visual dominance might be present in healthy younger adults, the observed frequencies of visual versus auditory balance should be used as baseline against which to compare the age-related influences on sensory profile and associated multisensory benefits within healthy older individuals. More generally, it will be important for future research to establish reliable and practical ways of assessing sensory dominance in order to determine its precise age-dependency and clinical applicability. Nonetheless, one major benefit of a simple detection task like the one used here is its emphasis on low-level processes without a reliance on either memory- or speech- related functions that are likely adversely affected by both healthy as well as atypical aging that can in turn impact sensory dominance. The present study provides here an essential proof of concept. The introduction of adaptive technologies could allow for refinement of the approach put forward here, particularly if such can be administered from a distance and in an automated fashion so as to use simple tasks (and the sensory and multisensory profiles that can be extracted from them) as potential, complementary screening tools.

The interplay between task-related and subject-specific (including age-related) influences on sensory dominance during multisensory processing is a domain of increased experimental inquiry^10,11,43^. Using a simple detection task with meaningless stimuli across all three groups, we were able to reveal that it is visual dominance that drives (1) enhanced multisensory benefits in healthy aging, and (2) lack thereof in abnormal aging. Notably, these low-level sensory and multisensory processes hold predictive value for age-related cognitive disturbances. Likewise noteworthy are data showing that sensory dominance and attentional preference can be readily dissociated (though a sufficiently long paradigm, lasting approximately 1 hour, is necessary to establish these preferences). That is, whether the auditory or visual modality had a lower detection threshold can be independent of which sensory modality had a greater influence on behaviour when performing a multisensory divided attention task^31^.

Perhaps the most exciting finding of our study is that a model combining the individual’s relative strength of sensory dominance and the relative strength of multisensory benefits, as measured in simple detection task, had diagnostic value in terms of MCI discrimination. In other words, on its own, neither the degree of one’s preserved low-level multisensory processing nor the relative extent to which one’s vision becomes impaired compared to the level of their hearing skills can on its own determine whether someone’s cognitive functioning can be categorised as characteristic of MCI versus HO. The two measures distinguished between HO and MCI only when combined, and the diagnostic validity of the resulting aggregate parameter was directly demonstrated with an ROC analysis. Notably, therein, its clinical validity, as indexed by the AUC value was within the range of those obtained from similar ROC analyses of the subscales of HVLT; a traditionally used MCI diagnostic tool. It is likewise noteworthy that the %MSI gain correlated with MMSE scores in MCI patients, but not with any HVLT score. This pattern is consistent with the idea that the multisensory processes indexed by the detection task introduced here may serve as a complement to other assessments and may measure distinct constructs^25,26^.

One could argue that it was the enhanced auditory processing found in the majority of healthy older and all MCI individuals that drives the diagnostic value of the aggregate sensory parameter. However, none of the raw responses, auditory, or visual or multisensory, for that matter, showed to be clinically diagnostic. It was the relative distance between one versus another sensory processing, combined with the associated benefit when two inputs are presented together, that was clinically diagnostic. The extent of sensory dominance and the magnitude of multisensory benefits were found to be negatively associated with each other (even when controlling for age), which is consistent with previous reports of stronger benefits in contexts where effectiveness of unisensory processes is comparable^38^. However, the fact that this relationship was evidenced only in participants exhibiting auditory dominance would suggest that matched effectiveness may not be the sole or the strongest contributor to multisensory interactions^44^. Our study reveals the diagnostic utility of the relationship between unisensory balance and multisensory benefits. Furthermore and more generally, our findings are consistent with the importance of multisensory processes in shaping higher-level cognitive function. Previously, our team has demonstrated that the strength of brain responses to a naturalistic object (e.g., image of cow) presented together with a meaningless stimulus in another sense (e.g., a beep) predicted memory for the same object presented subsequently on its own^45^. As in here, no similar relationship was found for unisensory responses. More recently, others have demonstrated that behaviourally assessed stronger cross-modal temporal perception abilities are associated with better skills in recognising audiovisual speech^35^. Our current results enrich these findings by highlighting the role of individual’s sensory profiles, which, while demonstrated as influencing multisensory processes already in young adults^30,31^, might come to play the primary role in alternating multisensory processes especially later in life. This notwithstanding, as to our knowledge this is the first demonstration of diagnostic validity of a measure including relative distances between unisensory and multisensory audiovisual responses.

Our findings open the exciting possibility that the multisensory detection task could be a valuable complementary screening and assessment tool for MCI. Currently, MMSE is widely used across the world as the primary diagnostic screening tool for MCI^26^. Yet, its use is by no means unanimously supported or even sufficient to provide a full MCI diagnosis. The overarching uncertainly is strongly driven by the fact that memory problems associated with mild dementia and those stemming entirely from healthy age-related changes are painstaking difficult to distinguish. As a result, in the majority of cases, additional tests are employed, such as the HVLT and its multiple subscales (as used in our study). While, as already mentioned, neither of these instruments on their own can provide an unequivocal diagnosis, the choice of the primary diagnostic tool itself continues to be a topic of a heated debate^25–27^.

MMSE is certainly the most widely used and studied brief cognitive screening assessment for dementia. MMSE measures a variety of separate domains, including episodic recall, orientation skills, attention, sequencing, copying and naming. It is believed to be a “reasonable” diagnostic tool for dementia in conjunction with other assessments, with good test-retest reliability and internal consistency^26,27^. At the same time, it has several important limitations. For one, its diagnostic properties vary (cut-off point: <23 or 24, typically; sensitivity and specificity: 71–92% and 56–96%, respectively, according to a meta-analysis^46^). Second and relatedly, age, ethnic background and especially educational level influences the MMSE scores. With respect to the latter, MMSE provides false positives for individuals with <9 years of education as well as ceiling effects for cognitively impaired individuals who possess higher education^26,27^. Additionally, while it can arguably be used by non-specialists, it requires some familiarisation and training^27^. In contrast, another popular brief cognitive test, the HVLT, which we used concomitantly to the MMSE in our study, was developed as a quick (<5 minutes) tool to be administered by clinicians without specialist psychometric training. A recent systematic literature review suggests that, in both primary and secondary care, it has better psychometric properties than MMSE^27^. It does not have ceiling effects and is insensitive to educational effects^25^. Nevertheless, its sensitivity and in particular its specificity varies (80–96% and 68–89%, respectively). Its main limitation is that the diagnostic properties of HVLT remain to be extensively validated. Furthermore, it focuses on specific skills, especially verbal registration and recall abilities and, as such, the scope of the tested functions is limited, in this case mainly to memory. Overall, the MMSE seems to be a good assessment tool for the general, current cognitive state of the individual. In contrast, the HVLT is a better indicator of the person’s learning and memory-based functions. An emerging consensus seems to be that HVLT has a better sensitivity, especially in early or mild dementia, while the MMSE has better specificity^25,27^. However, both require some degree of a specialist training and are preferably administered in well-controlled, clinical settings. In these domains, the observed sensory parameters could offer a promising, in-home, robust complementary tool.

Aware of this continued debate, the second aim of our study was to explore whether there is an added benefit in MCI diagnosis of a multisensory detection tasks such as used here. Our reasoning was motivated on the one hand by the growing number of findings of differences in sensory profiles among older individuals and their potential role in altering multisensory processing^29,39,47^. As detection tasks (and likely other tasks) involve assessment of continuous measures of performance, when equipped with multisensory stimuli, they open the possibility of measurement and quantification of potentially fine-grained differences in the integrity of unisensory systems, as well as in the integrative processes operating on the information extracted within these pathways. We reiterate that this versatility of the multisensory detection task is important inasmuch as unisensory impairments alone were not clinically diagnostic, and research is rife with evidence for differences across brain and behaviour in processes engaged by unisensory versus multisensory processing^9,11^. On the other hand, and most notably, the perceptual task, as the one employed here, offers this rich information while being fast (<10 minutes), cheap (requiring only a laptop) as well as robust and easy to administer in patients’ homes by individuals who do not require extensive neuropsychological training. These characteristics make it a potentially extremely valuable assessment tool in the area of cognitive functioning in the older population, where it can be used for wide-range screening for early dementia; area, where such traditional, popular tests, such as the MMSE, fail^27^. Similarly, it could serve as a complementary assessment tool, together with more traditional, neuropsychological tests and questionnaires. One potential limitation of the detection task as used here is that there was no explicit calibration of the stimuli and their effectiveness. As such, the stimuli themselves may have been the main driving force in producing the observed profiles of sensory dominance (though we would remind readers that there was no reliable difference in unisensory processing in the HY). Such notwithstanding, the highly reliable negative correlation between multisensory gain and relative auditory dominance in conjunction with the fact that the ROC analysis was reliable only when based on the linear combination of these two metrics would suggest that any diagnostic utility should withstand variations in unisensory effectiveness; though this will naturally require empirical demonstration. One next step would certainly be to replicate this diagnostic validity of the multisensory detection task in a larger sample in a randomised clinical trial. This notwithstanding, we were already capable of demonstrating the diagnostic validity of the detection task at levels comparable with those characterising some of the subscales of the traditional neuropsychological tools, such as the HVLT.

While such an interpretation is rather speculative at this stage, we believe that the observed pattern of results is consistent with the importance of the cholinergic system and the wide range of changes it induces across both the brain and cognitive capabilities as a result of healthy aging and neurodegenerative processes. The cholinergic system is primarily responsible for supporting top-down inhibitory processes across multiple levels of the brain. The efficacy of the cholinergic system declines in healthy aging, and is disrupted by abnormal aging processes, such as those underlying MCI or AD^17^. Consistently with the importance of this system for supporting the processes ensuring the integrity of higher-level capabilities, such as top-down attention or memory^17^, healthy older and MCI individuals perform differently on the MMSE, aimed to detect even minor impairments in general cognitive functioning and the HVLT that captures more specific impairments in learning, delayed recall and recognition memory. Crucially, the age-based decline and abnormal disruption of the integrity of the cholinergic system fits well with the pattern of multisensory benefits observed in our study. That is, while the healthy older individuals demonstrated overall enhanced multisensory benefits compared to healthy younger individuals, no similar advantage was observed for older individuals with MCI. The fine differences in responding to multisensory stimuli may well reflect the minutiae impairments in the functioning of the cholinergic system, which, when not detected early in time, could lead to more major disruptions in brain and cognitive functioning, such as those found in AD^22^. Our results clearly demonstrate the need for carefully designed longitudinal studies that will clarify the relationship between changes in the cholinergic system functioning, multisensory processing and the differential patterns of cognitive functioning in healthy and abnormal aging. Low-level perceptual processing may offer both highly sensitive and specific early diagnostic tools and provide an opportunity for targeted, possibly cholinergically mediated, interventions.

## Methods

### Participants

A total of 123 participants partook in the experiment, recruited from three different populations: 51 healthy young (HY) adults, 49 healthy older (HO) adults and 23 older MCI adults. HY were recruited via the university’s subject pool (participants were offered course credit for participation) and via word of mouth. HO were recruited through private care homes, the University of the Third Age, and word of mouth. Five participants (1 HY, 3 HO, 2 MCI) were excluded because their accuracy on auditory trials in the detection task was <90%, indicating a potential hearing loss. Further 3 HO and 3 MCI were excluded because their performance was excessively slow (>4 SDs above group mean). Three HY were excluded due to non-compliance with instructions. Finally, 7 older adults with an MMSE score of 27 (between the criteria for ‘healthy’ and ‘MCI’; detailed below) were excluded. There were 47 HY, 35 HO, and 18 MCI in the final sample (see Table 1). The research was reviewed and approved by the Department of Psychology Ethics committee of the University of Westminster and met the British Psychological Society ethical standards. All participants provided written, informed consent for their participation.

### Neuropsychological Assessments

A brief neuropsychological battery was used to capture learning and memory deficits. The Hopkins Verbal Learning Test (HVLT)^48^ was administered to assess learning (HVLT-learning), delayed recall (HVLT-delayed) and recognition memory (HVLT-recognition) in three stages, to provide measures of verbal encoding, retrieval and discrimination. The assessment requires participants to learn and immediately recall a list of 12 words that is presented verbally in 3 trials, to provide a total learning score (out of a total score of 36). The participants are asked to recall the list of 12 words after a delay of 20 minutes, to provide a total delayed recall score (out of a total score of 12), and to distinguish between presented words and distractor words, in order to provide a measure of recognition memory (out of a total score of 24). Lastly, the percent retention was calculated as the best performance on trial 2 or 3 minus the delayed recall score, multiplied by 100.

The Phonemic and Semantic Verbal Fluency Test^49^ was administered to provide an indication of attentional and effortful strategic executive processes that are associated with the frontal lobe function. Participants are asked to produce as many exemplars beginning with specific letters (F, A and S) in 60 seconds, while avoiding pronouns, inflections and repetitions. The semantic fluency task captures the ability of the participant to generate exemplars from a given category of animals, which captures the integrity of the semantic network and is indicative of the temporal lobe function.

The Mini Mental State Examination (MMSE)^50^ was administered in order to classify older adults into those with healthy-memory (HO) and impaired-memory (MCI) groups. The MMSE is a tool that is used by clinicians such as GPs or neuropsychologists to help them diagnose and assess dementia. Many traditional tests of the MMSE use a cut-off of ≥23 as ‘normal’ functioning, but many researchers have argued that this incorporates individuals with MCI. There is currently no agreed cut-off for defining ‘healthy’ functioning^51^. Based on clinical investigations of healthy aging and dementia^52,53^, we defined HO individuals as those with a MMSE ≥ 28, and MCI individuals as those with a MMSE ≥ 20 and ≤26. The researcher was supervised by a qualified clinical psychologist and neuropsychologist, and it was made clear that this tool was being used for research purposes and not as a clinical diagnostic tool.

The National Adult Reading Test (NART)^54^ was used to provide an estimate of premorbid crystallised intellectual function that remains stable across the lifespan and is typically preserved across a range of pathological conditions. Participants were asked to read a list of 50 irregular words aloud. Scores were marked in errors, which enabled the subsequent calculation of predicted full scale IQ.

### Multisensory Detection Task

Participants completed the task on a Dell Latitude E6420 laptop. The experiment was generated using Eprime-2 software (Psychology Software Tools; www.pstnet.com), and the data later merged and extracted into SPSS version 23 for analyses. The experiment presented visual, auditory, or simultaneous auditory-visual stimuli (V, A, AV, respectively). The visual stimulus was a black circle, subtending 7.9° and presented centrally against a white background. The auditory stimulus was a 1000 Hz sinusoidal pure tone presented via the built-in speakers of the laptop. The volume of presentation was individually adjusted to a comfortable level before completing the task. A Casella Cel Dawe D-1422c sound meter recorded the sound level at the ear for most participants (31 HY, 28 HO, and 17 MCI). There was no significant difference in the sound volume across participant groups (HY mean: 70.5 ± 9.8 dB; HO mean: 72.3 ± 8.8 dB; MCI mean: 71.8 ± 8.2 dB; F_(2,73)_ < 1, p > 0.1).

We chose basic stimuli, as these have low likelihood of being influenced by memory-based and aging-based processed. While not explicitly nor effortfully matched in the HY group, RTs to the visual and auditory stimuli were in fact statistically indistinguishable in this age group, as purveyed when analysing data from the first few participants. Our choice of such a prescribed range of stimuli was also guided by prior works showing that sensory dominance and attentional preference are dissociable aspects of stimulus processing^31^. Specifically, they showed that any effect of attentional preference, expected to be influenced by stimulus heightened salience, cannot be explained simply by detection threshold, or vice versa.

Participants completed 4 blocks, each containing 50 trials. Within each block, each stimulus condition (V, A, and AV) was delivered 15 times, in addition to 5 catch trials with no stimulus. The stimulus duration was 100 ms with a pseudo-randomized inter-stimulus interval (ISI) of 2500–3500 ms to prevent anticipation in combination with the above mentioned catch trials. Inclusion of an individual’s data required ≥ 90% detection accuracy on all stimulus conditions. Within an individual, all reaction times (RTs) below 200 ms and more than 2 standard deviations above the mean RTs were excluded from analysis. On average across all tasks (standard deviation indicated), 0.6 ± 0.7%, 0.5 ± 0.7%, and 1 ± 1.2% of trials (ranges 0–2.67%, 0–3%, and 0–3.67%) were excluded from analyses from HY, HO, and MCI, respectively.

### Procedure

Participants engaged in a single session conducted in a quiet private room located at the University of Westminster, in participants’ homes, or at a private residential care home. The locations were selected for the ease and convenience of individual participants; again with the objective of determining to what extent a quick and low-cost task such as simple detection would be effective in pre-screening for risk of MCI. Participant demographics were collected before administering the Edinburgh Handedness Inventory^55^. For the neuropsychological tasks, the HVLT-R learning task was conducted first, followed by the Phonemic and Semantic Verbal Fluency Test. Participants were then told that they would be presented with either black circle, a beep or both of these stimuli together. Whilst focusing on a central fixation cross, participants were told to simply press the spacebar on the laptop keyboard with their dominant hand when they detected any of the three conditions. Participants were not required to discriminate between the stimulus conditions. The whole task lasted approximately 10 minutes. Following this, the NART was administered to participants, after which all older participants completed the MMSE and, finally, the HVLT-R delayed recall and recognition tasks were completed.

### Analysis design

Building on previous work, analyses of performance on the detection task focused on identifying differences in unisensory and multisensory processing across the three groups, with a particular focus on group differences in sensory dominance profiles and associated relative magnitude of multisensory RT benefits.

To assess the clinical viability of both multisensory RT gains and relative auditory dominance, we employed the ROC (receiver operating characteristic) curve analysis, a fundamental tool for diagnostic test evaluation. In the ROC curve, the true positive rate (Sensitivity) is plotted against the false positive rate (1-Specificity) for different cut-off points of a chosen parameter. Each point on the ROC curve represents a sensitivity/specificity pair corresponding to a particular decision threshold. The area under the ROC curve (AUC) is a measure of how well a parameter can distinguish between two diagnostic groups (diseased/normal). Statistically, ROC analysis is a logistic regression, where the tested, interval parameter is used a predictor of a binary outcome variable, in this case, healthy (value 0) versus clinical group (value 1). To assess collinearity we used the Variance Inflation Factor (VIF), which is calculated as 1/(1 − R^2^). The VIF in the case of multisensory gain and sensory dominance was 1.6, which is not considered as high^56,57^.

### Data availability

The datasets generated during and/or analysed during the current study are available from the corresponding author on reasonable request.

## Electronic supplementary material

Supplemental Materials
