# Supplementary material for: Sensory dominance and multisensory integration as screening tools in aging
Source: Sci Rep. 2018 Jun 11;8:8901. doi: 10.1038/s41598-018-27288-2 (PMC5995929; doi:10.1038/s41598-018-27288-2)
Supplement: Supplementary file 1 — Supplemental Materials [file 41598_2018_27288_MOESM1_ESM.docx]

**Supplemental Materials**

**Sensory dominance and multisensory integration as screening tools in aging**

Micah M. Murray, Alison F. Eardley, Trudi Edginton, Rebecca Oyekan, Emily Smyth, and Pawel J. Matusz

**Supplemental Results**

***Neuropsychological tasks***

Supplemental Table S1 reports the mean scores (standard deviations indicated) across several of the neuropsychological tasks for each participant group as well as the F-test results and post-hoc contrasts. The HO participants performed better on the Phonemic verbal fluency task, HY and HO performed equivalently on all other tasks. However, the general pattern of performance across the data demonstrated a pattern of poorer learning, delayed recall and recognition memory performance for MCI adults.

***Detection task***

Performance on the detection task was at near-ceiling levels in all groups (>97.5% correct in all groups and stimulus conditions). There was no evidence that accuracy differed across these factors (group × condition interaction: F<1). We also examined the rate of commission of false alarms, which averaged 0.78%, 0.43%, and 2.5% across the HY, HO, and MCI groups, respectively. Given the skewness of these data we performed a non-parametric 1-way ANOVA (Kruskal-Wallis test) after arcsin transformation, which indicated that these false alarm rates differed across groups (p=0.020). Finally, we calculated Pearson correlation coefficients between accuracy on a given stimulus condition and false alarm rates, which were significantly negatively correlated in all cases (p’s<0.015).

Multisensory gain, as a percentage change in RTs, was calculated for each participant using the following formula:

$$100 \times\frac{({RT}_{Unisensory}-{RT}_{Multisensory})}{({RT}_{Unisensosry})}$$

where RT_unisensory_ is the faster mean RT between the auditory and visual conditions and RT_multisensory_ is the mean RT to the auditory-visual condition. Mean gain values are displayed in Figure 1B. Because of the zero count of visually-dominant MCI adults, two separate ANOVAs were run also on this measure of multisensory gain. The first was a 2 (Group) x 2 (Dominance) independent-design ANOVA comparing younger and healthy older adults. Overall, visually-dominant individuals exhibited larger multisensory gains than auditory-dominant individuals, irrespective of age group (main effect of sensory dominance: F_(1,78)_=12.2, *p*=.001, η_p_^2^=0.93). There was no main effect of group or an interaction, both *p’s*>.1). In turn, gains in the auditory-dominant individuals were analysed using a 1-way ANOVA, which revealed that multisensory gain across HY, HO, and MCI were comparable in size (F_(2,69)_=1.1, *p*=.34, η_p_^2^=0.24).

***Race Model Inequality***

In order to test whether the multisensory facilitation of response times described above exceeded predictions based on probability summation, data were analysed according to Miller’s race model inequality^1^. Briefly, the cumulative distribution functions (CDF) of the RT data for each condition are compared after dividing the data into bins of 10% width. Specifically, the CDF for AV stimuli is compared with the sum of the CDFs for A and V stimuli minus their product, so as to account for joint probability. Positive values of this comparison are indicative of facilitation beyond probability summation. Such facilitation was observed across groups and dominant sense over the fastest 10-30% of the RT distribution, for the visually dominant healthy young and old participants (Supplemental Figure 1B). We then compared the extent of race model violation over this portion of the RT distribution with a two-way RT bin x Group mixed-model ANOVA for auditory dominant and visual dominant groups, respectively. For the auditory dominant group, there was a main effect of RT bin (F_(2.48,171.42)_=20.88; *p*<.001; η_p_^2^=1.00). This was unsurprising, given there was no multisensory gain in the first bin, and simply indicated a higher multisensory gain for some bins than others. Neither the main effect of group nor the interaction was significant (both *p’*s>.1). Nevertheless, as displayed in Figure 1C (left panel) for all groups multisensory gain was significantly above chance for the largest and second largest time bins (HY and HO, all *t*’s≥3.92, *p*’s≥.01; MCI, both *t*’s≥2.59, *p*’s<.05). Crucially, across the two visually dominant groups, there was a main effect of group (F_(1, 26)_=5.43; *p*=.028; η_p_^2^=.61), suggesting generally larger race model violations in HO than HY (Figure 1C, right panel). There was again a main effect of RT bin (F_(2.40,62.44)_=19.07; *p*<.001; η_p_^2^=1) and no two-way interaction (p>.1). For both HY and HO, multisensory gains were significantly larger than chance for the largest and second largest time bins (all *t*’s≥4.51, *p*’s≤.01).

**Supplemental References**

1. Miller, J. Divided attention: evidence for coactivation with redundant signals. *Cogn. Psychol.* **14,** 247–79 (1982).

**Supplemental Table S1.** Cognitive performance across participant groups. Mean scores and standard deviations are indicated as well as F-test results. Post-hoc tests, subsequent to a significant 1-way ANOVA, are listed only in cases where these were statistically significant (p<0.05).

| Variable | HY | HO | MCI | F-test results | Post-hoc tests |
| --- | --- | --- | --- | --- | --- |
| Verbal Fluency (phonemic) | 36.77 (10.62) | 47.66 (16.21) | 33.89 (13.86) | F_(2,97)_=8.97; p<0.001; η_p_^2^=0.16 | HO>HY  HO> MCI |
| Verbal Fluency (Semantic) | 20.23 (4.56) | 21.20 (5.01) | 15.50 (3.52) | F_(2,97)_=9.83; p<0.001; η_p_^2^=0.17 | MCI<HY  MCI>HO |
| Hopkins Verbal Learning Test (learning) | 24.06 (4.81) | 25.03 (4.88) | 19. 94 (5.25) | F_(2,97)_=6.24 p=0.003; η_p_^2^=0.11 | MCI<HY  MCI>HO |
| Hopkins Verbal Learning Test (delayed recall) | 9.04 (1.93) | 9.06 (2.09) | 5.78 (3.57) | F_(2,97)_=14.38; p<0.001; η_p_^2^=0.23 | MCI<HY  MCI>HO |
| Hopkins Verbal Learning Test (recognition memory) | 22.38 (1.98) | 22.53 (1.26) | 20.94 (1.73) | F_(2,97)_=5.74; p=0.004; η_p_^2^=0.11 | MCI<HY  MCI>HO |

**
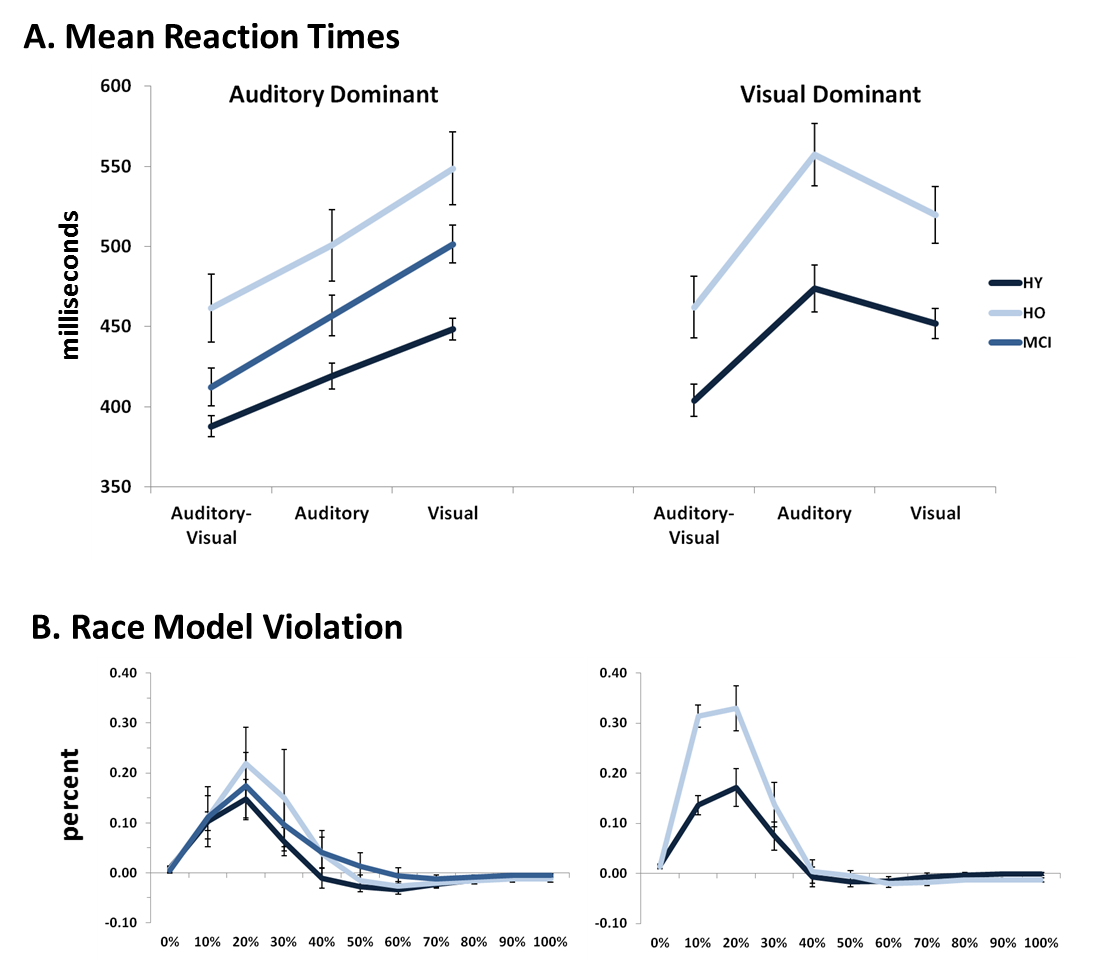
**

**Supplemental Figure S1.** Detection task performance separated according to sensory dominance. **A**. Mean RTs in the task, presented separately for auditory (left panel) and visually dominant (right panel) participants, as a function of stimulus condition (auditory-visual, auditory, visual) and participant group (HO, HY, and MCI). **B.** Results of the race model violation analyses, displaying successive bins in the normalised distributions of RTs across the three participant groups, presented separately for auditory (left panel) and visually dominant (right panel) participants for the three participant groups (HO, HY, and MCI). Notes: In all plots error bars indicate standard error of the mean. There were no visually dominant MCI participants.
